# Levers and Barriers to Vaccinate against COVID-19 in the Multicultural Context of French Guiana: A Qualitative Cross-Sectional Survey among Health Care Workers

**DOI:** 10.3390/vaccines9111216

**Published:** 2021-10-20

**Authors:** Maylis Douine, Sibylle Granier, Kepha Brureau, Jacques Breton, Céline Michaud, Mélanie Gaillet, Camille Agostini, Mathilde Ballet, Mathieu Nacher, Audrey Valdes, Philippe Abboud, Antoine Adenis, Félix Djossou, Loïc Epelboin, Nicolas Vignier

**Affiliations:** 1 Centre d’Investigation Clinique Antilles Guyane, CIC Inserm 1424, DRISP, Centre Hospitalier de Cayenne, Av des Flamboyants, 97300 Cayenne, France; graniersibylle@gmail.com (S.G.); k97.brureau@gmail.com (K.B.); mathieu.nacher66@gmail.com (M.N.); antoine.adenis@ch-cayenne.fr (A.A.); vigniernicolas@yahoo.fr (N.V.); 2 TBIP (Tropical Biome and Immuno Physiopathology), U1019-UMR9017-CIIL Centre d’Infection et d’Immunité de Lille, Institut Pasteur de Lille, CNRS, Inserm, Université de Guyane, Campus de Troubiran, 97337 Guyane Française, France; felix.djossou@ch-cayenne.fr (F.D.); epelboincrh@hotmail.fr (L.E.); 3 Département Universitaire de Médecine Générale Montpellier-Nîmes, Université de Montpellier, 163 rue Auguste Broussonnet, 34090 Montpellier, France; 4 Département Universitaire de Médecine Générale, Campus de la Fouillole, Université des Antilles, BP 145, 97145 Pointe-à-Pitre, France; 5 Union Régionale des Professions de Santé—Médecins Libéraux de Guyane, Av des Flamboyants, 97300 Cayenne, France; jacques.14@icloud.com; 6 Centres Délocalisés de Prévention et de Soins, Centre Hospitalier de Cayenne Andrée Rosemon, Av des Flamboyants, 97300 Cayenne, France; celine.michaud@ch-cayenne.fr (C.M.); melanie.gaillet@ch-cayenne.fr (M.G.); 7 Centre Hospitalier Ouest Guyanais, Av Paul Castaing, 97320 Saint Laurent du Maroni, France; c.agostini@ch-ouestguyane.fr; 8 Agence Régionale de la Santé de Guyane, Av des Flamboyants, 97300 Cayenne, France; mathilde.ballet@ars.sante.fr; 9 Hygiene Department, Centre Hospitalier de Cayenne Andrée Rosemon, Av des Flamboyants, 97300 Cayenne, France; audrey.valdes@ch-cayenne.fr; 10 Unité des Maladies Infectieuses et Tropicales, Centre Hospitalier de Cayenne Andrée Rosemon, 97300 Cayenne, France; philippe.abboud@ch-cayenne.fr; 11 Institut Pierre Louis d’Épidémiologie et de Santé Publique, Inserm UMR 1136, Department of social Epidemiology, IPLESP, Sorbonne Université, 27 rue de Chaligny, 75012 Paris, France; 12 UFR SMBH, Faculté de Médecine, Université Sorbonne Paris Nord, 93000 Bobigny, France

**Keywords:** COVID-19, vaccination, French Guiana, willingness, barriers, multiculturalism, information, qualitative research

## Abstract

In French Guiana, a French overseas territory in South America facing a fourth wave of COVID-19, vaccination coverage is very low, both in the population and among health care workers (HCWs). Vaccine hesitancy concerned 35.7% of the latter in early 2021. The objective of this complementary study is to understand barriers and levers and to adapt messages to increase vaccination coverage among HCWs. We conducted a regional cross-sectional survey of HCWs with a questionnaire containing open-ended questions exploring factors associated with vaccine hesitancy and the needs to adapt the vaccination campaign in French Guiana. The discourses were analyzed using a qualitative approach based on grounded theory, with open coding of data by themes and construction of abstract categories. The analysis of the 357 responses collected from January to March 2021 reveals several trends. The ethical aspect of the HCWs’ role emphasizes the importance of getting vaccinated themselves (to protect patients, to set an example...) and of vaccinating as many people as possible, including the most geographically or socially distant, such as undocumented migrants. However, some HCWs remain suspicious of the vaccine with concerns over the efficacy and side effects, of health institutions, and of the pharmaceutical industry. The role of fake news circulating on social networks has been widely discussed. Efforts to explain and convince HCWs must be continued in French Guiana using the identified levers to improve the acceptability of vaccination.

## 1. Introduction

France is known for having one of the highest rates of vaccination hesitancy in the world [1]. In French Guiana, a French overseas territory in South America, vaccine coverage is even lower, including among health care workers (HCWs). In late May 2021, after five months of a vaccination campaign, only 7.9% of the population was fully vaccinated, whereas coverage in mainland France was 16.2% [2]. In August 2021, the difference is even more striking: 16.6% versus 64.5% [3,4].

Although not accurately counted, the number of COVID-19 cases among HCWs has been significant in French Guiana with occasional secondary cases among their patients. In January–March 2021, the first results of a survey on vaccine hesitancy in HCWs in French Guiana showed that 65.6% of them intended to be vaccinated against COVID-19 or were already vaccinated against COVID-19, with a higher proportion among physicians, then nurses, followed by other paramedical professions [5]. This raised two issues. First, HCWs are in frequent contact with infected patients and are therefore at greater risk of contracting COVID-19 which can lead to substantial morbidity and absenteeism from work at a time when the healthcare system is already overwhelmed by a deficit in infrastructures and human resources [6,7]. Furthermore, if positive, these HCWs can transmit the disease to other patients. Second, willingness to be vaccinated against COVID-19 for oneself correlated with recommendation of the vaccine to relatives and patients. In the initial results of the HCWs study, 93.0% of the vaccinated or those intending to do so were willing to recommend the vaccine to their relatives and 91.2% to their patients, whereas these percentages were 21.5 and 35.3% for the unsure [5].

French Guiana is located in the north of the Amazon forest, between Brazil and Suriname. This region of 84,000 km^2^ is sparsely populated with 300,000 inhabitants, mainly living on the coast where the three main cities and the three hospitals are located. Health care for the rest of the territory, accessible only by the air or by the river, is delivered by remote prevention and care centers (RPCC), especially on the Maroni and Oiapoque border rivers. The history and the successive migration waves led to great ethnic and cultural diversity, with more than 20 languages spoken commonly in French Guiana, such as French, Creole, Brazilian, Native American languages, Maroon languages, etc. [8,9,10]. All these geographical and cultural specificities must be considered in the organization of the vaccination campaign in French Guiana.

Although statistics on vaccine hesitancy are important, they sometimes fail to deliver the intricacies of individual motivations, which are keys to understand vaccination obstacles and levers among health professionals. We therefore conducted a qualitative analysis of HCW’s discourses to gain an in-depth understanding of HCWs’ attitudes towards the COVID-19 vaccine and the opinions of the HCWs on how the vaccination campaign should be adapted to the specific context of French Guiana.

## 2. Materials and Methods

### 2.1. Study Design and Population

From 22 January to 26 March 2021, a regional descriptive cross-sectional survey was conducted among HCWs in French Guiana. All HCWs working in private or public facilities and agreeing to participate were eligible. At the time of the inclusions, vaccination was accessible to all HCWs, general population over 50 years old and persons with comorbidities [2]. The only available vaccine was the mRNA vaccine BNT162b2 from BioNTech-Pfizer^®^, Mayence, Germany, New York, NY, USA.

### 2.2. Sampling and Procedure

The study was conducted using a self-administered semi-structured online questionnaire with multiple choice and open questions. An online platform (https://www.wepi.org/ accessed on 10 January 2021, Epiconcept^®^, Paris, France) with certified server to host personal health data was used to conduct the survey. All possible and available diffusion lists were used to reach all HCWs across French Guiana: from the head of the three main hospitals and of the 17 RPCC in isolated villages, regional union of liberal physicians and nurses, regional health agency (weekly letter), professional WhatsApp^®^ groups and mailing lists. HCWs were contacted by phone or physically by medical residents. An anonymous paper version of the questionnaire was also made available in RPCC for those having internet access difficulties and then entered in the Wepi platform. A reminder was posted via a QR code on the desktop of all computers at the main hospital in Cayenne as well as by the managers of the various departments.

### 2.3. Data Collection

Data were obtained directly from participants. No identifying data was requested. The questionnaire contained socio-demographic data, multiple-choice questions about the representation of vaccines in general and of COVID-19 vaccine in particular, as well as their willingness to get vaccinated and its associated factors. Additional data were added such as the origin of health professionals, activity and mode of practice. At the end of the questionnaire, two open questions were asked without answer size limits: “Please detail the reasons why you are for or against vaccination against COVID-19” and “Can you name three things essential to consider regarding COVID-19 vaccination in French Guiana?” (Supplementary Material). The first qualitative question aims assessing the representation of the COVID-19 vaccine and the second the obstacles and levers to vaccination in regards with French Guiana territorial specificities.

### 2.4. Statistical Analysis

Anonymous data collected through the Wepi platform were extracted in an Excel format. The study was based on mixed methods. The quantitative data were described using standard tests with Stata^®^ 15.1 software (StataCorp, College Station, TX, USA). Detailed descriptive results and factors associated with the willingness to get vaccinated by HCWs were presented in a previous publication [5].

The HCWs’ views and discourses from the two open questions, which were transcribed, were analyzed by two public health physicians and researchers using framework analysis according to the standards of reporting qualitative research [11]. The qualitative approach was based on grounded theory, with reading the discourses, open coding of the data by themes related to the research questions, and construction of abstract categories inductively [11]. The number of respondents was sufficient to reach saturation of the themes studied. The data were summarized into an analytic framework. The data were double-coded by MD, KB, and SG.

Word clouds were used to represent the textual data as a concise visual summary of the main words used by the participants [12]. The word clouds were generated using the free online software www.wordclouds.com (access date 1 July 2021) based on the frequency of word occurrences after correcting for plurals, grammar, and spelling.

### 2.5. Ethics and Regulation

Discourses were collected in a strictly anonymous manner with the participants’ consent collected online on the accredited website wepi.org. The collection of data has been subject to the individual information of participants, a privacy impact analysis, and the study online deposit on the French Health Data Hub platform in accordance with the French and European General Data Protection Regulations. No ethical approval was required.

## 3. Results

Among the 579 participants included during the two-months study, 357 answered the open questions with qualitative material: 242 women and 115 men. Table 1 describes the study population.

The results of inferencing and the thematic content analysis can be classified into the three main topics above.

### 3.1. Motivation to Be Vaccinated

**Figure 1:**
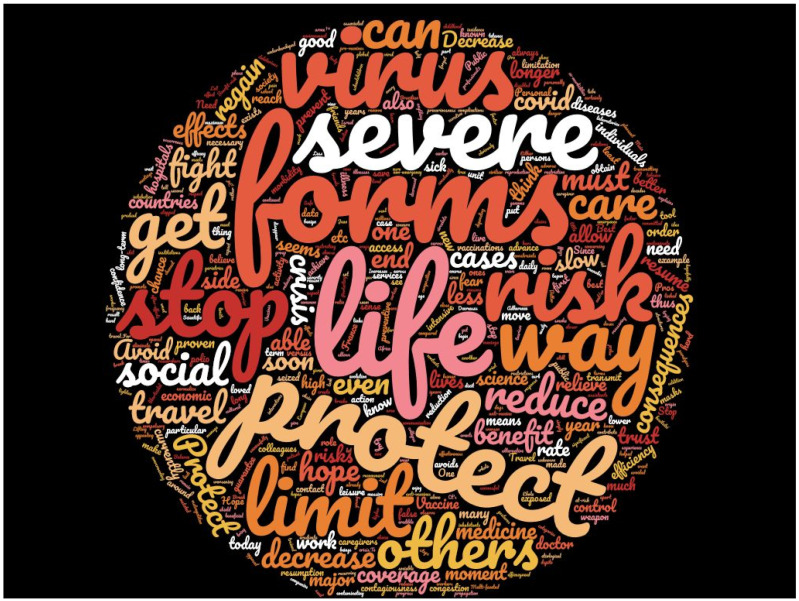
Word cloud from health care workers’ responses to open-ended questions in favor of COVID-19 vaccination.

Vaccination was perceived by a significant proportion of HCWs as a major solution to fight the epidemic (“Prevention is better than cure”), a key tool, even if many specified that no alternative exists in their opinion. These HCWs trusted science, the effectiveness of the vaccine, and the low risk of adverse events. For many respondents, vaccination reduced the number of cases and therefore the impact on the health care system, the number of serious cases, and therefore the burden on intensive care units and the deaths. Vaccination also reduced the long-term consequences of COVID-19 (so-called “long COVID-19”). For these professionals, the reduction of the burden on the health care system would allow them to resume better management of all other patients, particularly those with chronic conditions. Being vaccinated as a professional allows them to “set an example”, not to contaminate their patients or their vulnerable relatives or colleagues, and to participate in a collective immunity. Many HCWs hoped that large-scale vaccination would allow a “return to normal life”, to “get out of the health and economic crisis”. This would be materialized by the lifting travel restrictions locally, but also outside French Guiana, by restarting leisure activities, by reducing the psychological impact of the lock-down, and even for some being able “not to wear the mask”. Some felt personally at risk (having risk factors) or had been struck by patients in intensive care and therefore wished to be vaccinated to protect themselves from a severe form. The verbatim of the caregivers adhering to the vaccination are represented in Figure 1.

### 3.2. Barriers to Vaccination

On the other side, a significant portion of caregivers are defiant about the vaccine. The main barriers mentioned for vaccination were the lack of knowledge about the vaccine against COVID-19 in terms of:

(i) Efficacy: clinical trials were considered too short, with a lack of hindsight on the efficacy of vaccines against circulating variants-of-concern (VOC) and of interest (“not worth it if a mutant virus is emerging”, “the virus mutates quickly, vaccination will always lag behind the mutations”), and on the short-lived immunity;
(ii) Reduction of transmission: if the vaccine does not allow for a minimum removal of barriers measures, then its value is limited, according to a number of respondents;
(iii) Potential adverse effects: the lack of hindsight on the possible long-term consequences, the adverse events, the mechanism of the RNA vaccine (sometimes considered as “genetic manipulation”) led to a significant fear of the vaccine.

**Figure 2:**
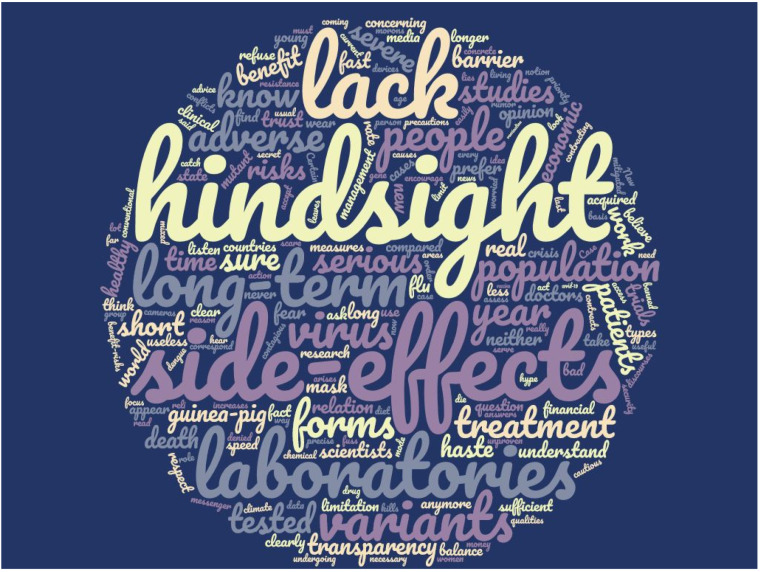
Word cloud from health care workers’ responses to open-ended questions against COVID-19 vaccination.

For other participants, the fear of adverse events was not linked to a lack of information, but considered it was an “established fact” that there are a high number of adverse events and/or allergies. This was in line with a distrust of pharmaceutical companies that were reported to only take into consideration their financial profits (“with such economic stakes, there is inevitably a risk of malpractice”), with a lack of transparency in their communication and in their relations with public institutions, especially the government, a situation considered to be the source of numerous conflicts of interest. Many HCWs had lost confidence in health institutions, considering that the government was responsible for “disinformation”. The disagreements and discordance between scientists, physicians and epidemiologists contributed to reinforce the distrust of the public. Some questioned the interest of vaccination given the epidemiological data considered reassuring in French Guiana, with a low incidence (at the time of the survey), a very low lethality and a young population with little risk of severe forms. These respondents believed that vaccination could be delayed in French Guiana to have more time to assess its effectiveness and side effects, or to vaccinate only those people at risk of severe forms. Several of the health professionals interviewed planned to wait for a little more time to assess the vaccine, as they considered themselves to be at low risk of severe disease and respected the barrier measures. The transcripts of HCWs not willing to get COVID-19 vaccine or having concerns are represented in Figure 2.

### 3.3. French Guianese Specificities to Be Considered for the Vaccination Campaign

#### 3.3.1. Specificities of French Guiana Making Vaccination against COVID-19 Appropriate

The majority of respondents considered that the vaccination campaign in French Guiana was appropriate because of:

(i) The high prevalence of risk factors for severe COVID-19 (hypertension, diabetes, obesity, HIV infection);
(ii) The “porous” borders with a risk of introducing VOC from neighboring countries;
(iii) The impact of the pandemic in economic, social, and food insecurity terms (hunger is common during COVID-19 in very precarious neighborhoods [13]);
(iv) And the weakness of the Guianese healthcare system. Indeed, according to many respondents, it is facing a chronic lack of human resources and infrastructure (especially in intensive care units), with a risk of rapid saturation.

#### 3.3.2. The Need for Accessibility to Vaccination in Several Ways

For most respondents, vaccination should be non-mandatory, free, and accessible. This accessibility was addressed in different ways. First, in terms of the target population: many felt that all persons living in French Guiana should be covered, including undocumented migrants (although a minority of people expressed a contrary opinion on this point). Then, the question of geographical accessibility was often put forward, with the importance of going towards isolated populations with outreach mobile teams going to isolated places on the border rivers (“Kampoes”) or in the shanty towns of the coastal cities, or for persons without transportation means. Some of the respondents specified that private professionals, non-government organizations, or work doctors should be associated with each other to bring the vaccines as close as possible to all concerned populations. Secondly, logistical challenges, particularly in the “interior”, was a source of concerns with, for example, the feasibility of a cold chain in the remote health care centers (some respondents thought that the vaccine should be stored at a temperature below 80°C until the RHCC), the hazards associated with transporting equipment within French Guiana, frequent power cuts, etc. Finally, several professionals expressed concern about the feasibility of the second dose, either in terms of the stock of vaccines available or in relation to the difficulty of following up the vaccinated “who will probably not return”. Some suggested telephone reminder systems.

#### 3.3.3. A Concern about the Communication around the COVID-19 Vaccine

The majority of respondents expressed concern about the information and communication about COVID-19 that is conveyed through media and social networks. Some respondents thought, or echoed people and patients expressing a strong distrust of the government, health authorities, medicine, or, more broadly, of anything that comes from mainland France, using strong words such as: “neocolonialism”, “guinea pig”, or “experimentation”.

Other respondents deplored the “fake-news” flooding social networks, perceived as very powerful, more so than traditional media such as television or radio, because many people do not use these media, but only the internet. Some people thought that the population does not understand the seriousness of the situation and does not feel concerned even if they have risk factors. Many respondents recommended transmitting clear information, intelligible by the population, in their own language, adapted “to the illiteracy that concerns a large part of the population”, in order to restore confidence and reassure “without infantilizing”.

The multicultural dimension of French Guiana was to be considered. Several emphasized the importance of health mediators, whose profession is developing in French Guiana. Others deplored the lack of promotion of the vaccine by local Creole leaders or by traditional chiefs “who are afraid to promote the vaccine for fear of reprisals” in case of side effects. “If there are only French Caucasians advocating for vaccination, there is no hope of convincing those who are resistant from other ethnicities or cultures, such as the Creoles”. The evangelical churches were perceived as an obstacle to vaccination for several respondents, by spreading false messages and encouraging people not to get vaccinated. The use of traditional plants/remedies as a preventive or curative measure against COVID-19 should be considered for some respondents “because it works” or because of the risk of drug interactions according to other people.

For some respondents, health professionals had a major role to play in motivating the population to get vaccinated by informing them as best as possible, adapting to the level of knowledge of patients, in a context of “low level of health education” according to some.

## 4. Discussion

These results show several trends. On the one hand, the ethical aspect of the role of HCWs emphasizes the importance of getting vaccinated themselves (to protect patients, to set an example...) and of vaccinating as many people as possible from a public health perspective, including those who are the most distant geographically or socially, such as undocumented migrants. However, some HCWs remained suspicious of the vaccine, health institutions, and the pharmaceutical industry. The role of fake news circulating on social networks was widely mentioned [14].

### 4.1. Limitations

The response rate is estimated at 40.4% for physicians and 10.8% for nurses. The representation of doctors and nurses is therefore satisfactory, although not perfect. Other paramedic staff are poorly represented. The sex ratio and age distribution are satisfactory [5].

Because the study was implemented by public health physicians, it is possible that responses were biased by socially acceptable or expected responses. In addition, HCWs opposed to COVID-19 vaccination may be less likely to participate and therefore underrepresented. Both biases could be reinforced by the small size of the HCWs community in French Guiana, leading to suspicion of data confidentiality. However, the characteristics of the respondent to the open questions were not different from the study population (Table 1).

### 4.2. Concerns about Side Effects and Distrust of Health Authorities

Fear of side effects is a known barrier of willingness to get vaccinated among health care workers elsewhere in Europe [15,16,17]. It often goes hand in hand with a lack of trust in laboratories and health authorities and could be improved with communication [18]. This fear and this distrust is correlated with the level of scientific education, with higher trust among physicians and midwives, followed by nurses and then other health workers [5,19]. Among the latter, willingness to be vaccinated is even lower than in the general French Guiana population, estimated at 49% in May 2021 [20]. For similar reasons, we are far from the 90% of intention to vaccinate among caregivers in Germany, for example [17]. The need for hindsight being put forward during this study carried out in early 2021 will perhaps be satisfied with time, allowing reassurance on the potential adverse effects of the vaccine. However, at the time of submission of this article, the vaccination coverage appears to remain low among HCWs in French Guiana, particularly among paramedics.

### 4.3. The Vaccine: A Way Back to Normal?

In contrast to this mistrust and reluctance to vaccinate, many HCWs are in favor of vaccination, particularly for altruistic reasons: to protect patients, to increase collective immunity, to be able to better care for patients with other pathologies, etc. [18,21]. These aspects were more emphasized than the personal benefits of protecting oneself and one’s relatives. Another very important factor is the hope of a return to normalcy: a return to cultural life and the possibility of moving around, a very important element in French Guiana, which is located on the other side of the Atlantic from Metropolitan France and is therefore subject to air flights. These aspects are in line with the motivations of the general population in several other studies [16,20].

### 4.4. HCWs’ Concern about the Feasibility of Vaccination throughout French Guiana and for All

HCWs expressed concern about the feasibility of offering vaccination to all persons living in French Guiana, due to logistical constraints, geographic isolation, and the many undocumented persons. Efforts have been made to address these challenges. Non-profit organizations (such as La Croix-Rouge, Médecins du Monde) have partnered with the health system (hospitals and health centers) to provide outreach vaccination in shanty towns and small isolated villages. Staff (HCWs, coordinators, and logisticians) have been dedicated to organize vaccination in isolated areas. Health mediators have been hired to build trust with the population and disseminate correct information. The intervention of health mediators has repeatedly proven to be useful in public health programs in French Guiana [22]. After more than six months of work, COVID-19 vaccination coverage rates are similar in the coastal center (18% complete vaccination schedule for all ages as of August 5, 2021), in the coastal region called “Savanes” (19%) and in the east, including the villages along the Oiapoque River (18%) [3]. This shows a homogeneity of access to vaccination for these populations. However, for the western region, including the city of Saint-Laurent-du-Maroni and the villages located on the Maroni River, coverage is much lower, estimated at 7% [3]. This difference seems to be more related to a negative perception of the vaccine than to a lack of access to vaccination [2]).

### 4.5. A Multiculturality to Be Taken into Account

For most respondents, addressing multiculturalism in French Guiana is crucial to conveying messages and building trust with the health system. It is interesting to note the great diversity of the birthplaces of health professionals (38 territories listed on four continents, see Table 1), which differs from the cultural diversity of the inhabitants of French Guiana [23]. This highlights the multiplicity of intercultural relationships. We commonly found a greater distrust of the vaccine and health authorities among health professionals and people born in French Guiana or the West Indies [5,20].

Recreating trust in this context, where the region’s history of French colonization, the slave trade, geographic distance from the capital, and the issues of migrant populations are intertwined, as well the COVID-19 vaccine seeming to be associated to the old continent or to the French authorities, is not easy. Much misinformation circulates, which is difficult to counterbalance by the prevention messages disseminated by the medical community, despite efforts to translate them into 19 local languages [19]. The role of trained health mediators and the partnership with community associations are therefore essential, in an “outreach” approach, starting from people’s knowledge and representations to provide accurate and appropriate information. The high level of vaccine mistrust observed in the field among community and traditional leaders is also an important challenge. Most of the actors come from the health system. The support of community and political leaders is still insufficient and would improve confidence in the prevention proposals of the health system. The involvement of traditional practitioners in the prevention of COVID-19, including vaccination, could also be beneficial as some respondents mentioned the importance of herbal medicine as a means of preventing the disease [24,25,26].

### 4.6. Findings Shared with the French Overseas Territories of the West Indies

French Guiana shares with the French West Indies the lowest vaccination rates in France and a common belonging to the Creole community. In French West Indies, there are also significant resistance to vaccines among health professionals, particularly among paramedics and firefighters. This reluctance seems to be correlated with majority opinions in the general population (distrust of “imported science”, attachment to traditional medicine and herbal medicine, etc.) and a mistrust of the health authorities and the French state [27].

### 4.7. A Fourth Wave Due to the Delta Variant in a Context of Low Vaccination Coverage

At the time of writing, French Guiana is facing a fourth wave of COVID-19 due to the Delta variant, while the third wave is not yet over, particularly due to low vaccination coverage, estimated at 16.6% (12 years and older) of the Guianese population at the end of July 2021 [3]. This proportion is much more lower than in mainland France (52.9% for the 12 years and older) [4]. Health care workers are exhausted after nearly 18 months of COVID-19 circulation resulting in overtime, cancelled vacations, and a diminished private life. However, the vaccination coverage of health care workers remains low: as of 13 August 2021, only 35% of staff in health and medical-social facilities in French Guiana have received at least one dose of COVID-19 [28]. This leads to increased contamination of health care workers (and therefore morbidity and absenteeism) and a risk of contaminating patients. A new law was voted in France at the beginning of August 2021, imposing compulsory vaccination to all health care workers from 15 September 2021. Reactions are likely to be contradictory in French Guiana given the existing defiance (several demonstrations and the mobilization of unions and associations have already taken place), but this decision is also likely to facilitate the choice of some of those who are hesitant. The penalty of losing salaries will be difficult to implement in French Guiana due to the lack of human resources in this isolated territory and the absence of possible reinforcement due to the overload of the health care system everywhere in France. The initial reassuring curve of COVID-19 incidence in French Guiana [29], and the argument put forward by some participants in this study that French Guiana would be “less at risk” due to the youth of its population no longer seems to hold up in the face of the more transmissible Gamma and Delta variants, which affect young patients more severely [30,31]. It is important to explain this new measure, with arguments to avoid deepening the existing divide with some caregivers. Thus, strategies to improve acceptability of this now mandatory vaccine should be adapted to the specific political, social, cultural, and economic context. Several initiatives along these lines are currently underway.

## 5. Conclusions

Some HCWs have concerns about efficacy and side effects and distrust the authorities, while others adhere to protect themselves and others. The results of this study have made it possible to adapt informative messages to increase vaccination coverage, but also to adapt the vaccination campaign in this French Amazonian territory. Efforts to explain and convince HCWs must be continued in French Guiana using the levers identified to improve the acceptability of vaccination. Given the resentment of health workers, listening, dialogue, and improving working conditions seem to be important prerequisites. Particular attention must be paid to avoid a drift towards community fractures.

## Figures and Tables

**Table 1:** Description of the study population, health care workers in French Guiana, January–March 2021.

			All Included		Answering Qualitative Questions		% Who Answered Qualitative Questions	*p*-Value (chi2)
			n	%	n	%	%	
N			579		357		61.6	
**Sex**								
	Female		393	67.9%	242	67.8%	61.6%	0.977
	Male		186	32.1%	115	32.2%	61.8%	
**Age**								
	18–34		187	32.3%	106	29.7%	56.7%	0.458
	35–49		198	34.2%	113	31.7%	57.1%	
	50–64		152	26.3%	107	30.0%	70.4%	
	≥65		42	7.3%	31	8.7%	73.8%	
**Profession**								
	Physician		220	38.0%	156	43.7%	70.9%	0.112
	Nurse		217	37.5%	121	33.9%	55.8%	
	Midwife		24	4.1%	17	4.8%	70.8%	
	Pharmacist		17	2.9%	11	3.1%	64.7%	
	Nurse supervisor		17	2.9%	7	2.0%	41.2%	
	Health care assistant		9	1.6%	1	0.3%	11.1%	
	Administrative		30	5.2%	7	2.0%	2.3%	
	Other *		45	7.8%	37	10.4%	8.2%	
**Place of birth**								overall *p*-value: 0.985
	**France**		488	84.3%	307	86.0%	62.9%	
		*French Guiana*	*115*	*19.9%*	*60*	*16.8%*	*52.2%*	*p-value for respondents born in France:* *0.515*
		*Mainland France*	*346*	*59.8%*	*230*	*64.4%*	*66.5%*	
		*French West indies*	*18*	*3.1%*	*13*	*3.6%*	*72.2%*	
		*other oversea French territory*	*9*	*1.6%*	*4*	*1.1%*	*44.4%*	
	**Europe**		17	2.9%	10	2.8%	58.8%	
		*Belgium, Netherlands, Spain, United Kingdom*						
	**America**		23	4.0%	12	3.4%	52.2%	
		*Argentina, Brazil, Colombia, Guyana, Peru, Suriname, Trinidad, Uruguay, USA, Venezuela*						
	**Africa**		39	6.7%	25	7.0%	64.1%	
		**North Africa**	11	28.2%	8	32.0%	72.7%	
		*Algeria, Morocco, Tunisia*						
		**Africa other-unspecified**	28	71.8%	17	68.0%	60.7%	
		*Benin, Cameron, Chad, Congo-Brazzaville, Democratic Republic of Congo, Guinea Bissau, Ivory Coast, Madagascar, Mali, Senegal, Togo*						
	**Asia**		4	0.7%	2	0.6%	50.0%	
		*India, Laos, Thailand*						

* health mediator, psychologist, laboratory technician, social worker, dietician, podiatrist….

## Data Availability

All the relevant data for our analyses are fully described in the paper and can be made available on request. All data used for the analysis are available on request from the corresponding author.

## Supplementary Materials

The following are available online at https://www.mdpi.com/article/10.3390/vaccines9111216/s1, File S1: Questionnaire on vaccinal intention of health care workers in French Guiana.
