# Supplementary material for: Levers and Barriers to Vaccinate against COVID-19 in the Multicultural Context of French Guiana: A Qualitative Cross-Sectional Survey among Health Care Workers
Source: Vaccines (Basel). 2021 Oct 20;9(11):1216. doi: 10.3390/vaccines9111216 (PMC8622149; doi:10.3390/vaccines9111216)
Supplement: Supplementary file 1 [file vaccines-09-01216-s001.zip › vaccines-1369269-supplementary.pdf]

QUESTIONNAIRE ON VACCINAL INTENTION OF HEALTH CARE WORKERS IN FRENCH GUIANA

Questions 2 to 5 are have been subject to a previous publication: Vignier et al, Vaccines, 2021. https://doi.org/10.3390/vaccines9060682

1. Socio-demographic data

1.1) How old are you?
- 18-34 years old
- 35-49 years old
- 50-64 years old
- 65 and over

1.2) You are :
- A man
- A woman

1.3) Where were you born?
- French Guiana
- Metropolitan France
- French West Indies
- Haiti
- Suriname
- Brazil
- Guyana
- Other:

1.4) What language do you speak most often in your family/home?
- French
- Guyanese Creole
- Haitian Creole
- West Indian Creole
- Portuguese
- Sranan tongo
- Amerindian language
- English
- Other: ....

1.5) How long have you lived in French Guiana?

1.6) What is your profession?
- Physician
  - If yes, what is your specialty :
- Nurse
- Caregiver
- Other, please specify :

1.7) What is your mode of practice?
- Liberal

- Health centre
- Hospital
- Other, please specify :

1.8) What is your municipality of practice :

- Cayenne agglomeration
- Other coastal commune
- Commune of the interior

1.9) In what year did you start practicing?

2. Opinion on vaccination in general

2.1) Do you support vaccination in general?

- Very favourable
- Somewhat favourable
- Somewhat unfavourable
- Not at all favorable
- Don't know
- Does not want to answer

2.1) Are you opposed to certain vaccinations in particular?

- Yes
  - If so, which ones?
- No
- Don't know
- Does not want to answer

2.2) Do you feel sufficiently informed about vaccination in general?

- Yes, it is
- Yes, rather
- No, not really
- No, not at all

2.3) Do you think you are up to date with your vaccinations?

- Yes
- No
- Don't know

2.4) Do you trust the information provided by the health authorities regarding vaccination in general (website, emails...)?

- Yes, it is
- Yes, rather
- No, not really
- No, not at all
- Don't know
- Does not want to answer

2.5) Do you fear adverse effects of vaccines in general?

- Yes, it is
- Yes, rather
- No, not really

- No, not at all
- Don't know
- Does not want to answer

2.7) Do you agree with the following information:

"When I vaccinate, it is not only to protect myself but also to protect my patient and others.

- Yes, it is
- Yes, rather
- No, not really
- No, not at all
- Don't know
- Does not want to answer

3. Concerning the flu vaccination

3.1) Have you been vaccinated against the Flu in 2019-2020?

- Yes
- No
- Don't know/don't remember

3.2) Have you been vaccinated against the Flu in 2020-2021?

- Yes
- No
- Don't know/don't remember

3.3) Do you think the flu vaccine is effective?

- Yes, it is
- Yes, rather
- No, not really
- No, not at all
- Don't know
- Does not want to answer

3.4) Do you think the flu vaccine can cause serious side effects?

- Yes, it is
- Yes, rather
- Not so much
- No, not at all
- Don't know
- Does not want to answer

4. Regarding Covid-19

4.1) Have you ever had the Covid-19?

- Yes, definitely (positive test)
- Yes, probably
- No
- Don't know

4.2) Have you been confronted with severe Covid-19 cases?

- Yes, among my patients

- Yes, among my circle of friends (family/work colleagues)
- No
- Don't know

4.3) Do you have any risk factors for severe disease other than your age?

- Yes
- No
- Don't know
- Does not want to answer

4.4) What is your level of concern about the Covid-19 epidemic?

- Very concerned
- Somewhat concerned
- Not really worried
- Not at all worried

4.5) What confidence do you have in the management of the health and economic crisis linked to COVID by the authorities?

- Total confidence
- Overall confidence
- Little confidence
- No confidence at all
- Don't know
- Does not want to answer

5. Regarding vaccination against Covid-19

5.1) Do you think you have been sufficiently informed about the Covid-19 vaccination?

- Yes, it is
- Yes, rather
- Not so much
- No, not at all
- Don't know
- Does not want to answer

5.2) Do you trust the information provided by the authorities on vaccination against Covid-19 (website, newsletters...)?

- Yes, it is
- Yes, rather
- Not so much
- No, not at all
- Don't know
- Does not want to answer

5.3) Do you have confidence in the laboratories that developed the first available vaccines?

- Yes, it is
- Yes, rather
- Not so much
- No, not at all
- Don't know

- Does not want to answer

5.4) How do you find out about vaccination?

- Official websites dedicated to vaccination, institutional newsletters
- Scientific articles to make my own opinion
- Discussions with trusted health professionals
- Discussions with family and/or friends
- Social networks, consumer sites
- Other, please specify :
- Don't know
- Not informed

5.5) Do you think that vaccines against Covid-19 are effective?

- Yes, it is
- Yes, rather
- Not so much
- No, not at all
  - Don't know
  - Does not want to answer

5.6) Are you particularly worried about certain types of vaccines?

- RNA vaccine, such as Pfizer or Moderna
- Viral vector vaccine, such as Astrazeneca
- Viral protein vaccine, like Sanofi and GSK
- Inactivated virus vaccine, such as Sinopharm *(in China for example)*
- DNA vaccine, like Inovio *(NC)*
- All types of Covid-19 vaccine
- No, not really
- No, not at all
- Don't know
- Does not want to answer

5.7) Are you concerned about serious side effects from these vaccines?

- Yes, it is
- Yes, rather
- No, not really
- No, not at all
- Don't know
- Does not want to answer

If yes, which ones?

5.8) Do you think there would be more adverse events with the Covid-19 vaccines compared to other existing vaccines?

- Yes, it is
- Yes, rather
- Not so much
- No, not at all
- Don't know

- Does not want to answer

5.9) Regarding vaccination against Covid19 :
  - I am already vaccinated against Covid19
  - I plan to get vaccinated for sure
  - I'm probably considering getting the vaccine
  - I am considering not getting vaccinated
  - I'm sure I won't be vaccinated
  - I haven't made my decision yet

5.10) What would motivate you most (or has motivated you) to get vaccinated? (*several answers possible*)
  - To be sure that the vaccine prevents as many severe forms of the disease as possible
  - Ensure that the vaccine minimizes transmission of the virus
  - To be sure that the vaccine is effective
  - Be sure that it has no or few adverse effects
  - Ensure that vaccine recommendations are made with groups of health professionals and scientists who have no vested interest in the vaccine producers
  - Wait for more vaccine recoil
  - Another reason (specify _____)
  - No argument is likely to change my mind

5.11) Do you think you would recommend vaccination to your relatives?
  - Yes, certainly
  - Yes, probably
  - No, probably not
  - No, certainly not
  - Don't know

5.12) Do you recommend vaccination to your patients?
  - Yes, it is
  - Yes, rather
  - No, not really
  - No, not at all
  - Don't know

5.13) If yes to the previous question, would you recommend vaccination against Covid-19 to a particular type of patient?
  - Yes

  If yes, which ones?
  - No

5.14) Of the last 3 patients you talked to about the covid vaccine, how many were planning to get vaccinated?"

6. Open questions

6.1) Please detail the reasons why you are for or against vaccination against COVID-19?

6.2) Can you name three things essential to consider regarding COVID-19 vaccination in French Guiana "?
